# A 33-Year-Old Man with a Facial Rash

**DOI:** 10.1371/journal.pmed.0010017

**Published:** 2004-11-30

**Authors:** John Fleming, William A Lynn

## Abstract

A learning module based around the case of a man with ezema who presents with a rash around his ear and eye. Test your knowledge with our online quiz

## DESCRIPTION of CASE

A 33-y-old man presented to his primary care practitioner with a rash beginning around the left ear and spreading into the periorbital region. He was known to have eczema (atopic dermatitis), and the diagnosis of secondary bacterial infection and periorbital cellulitis was made.

**Figure 1:**
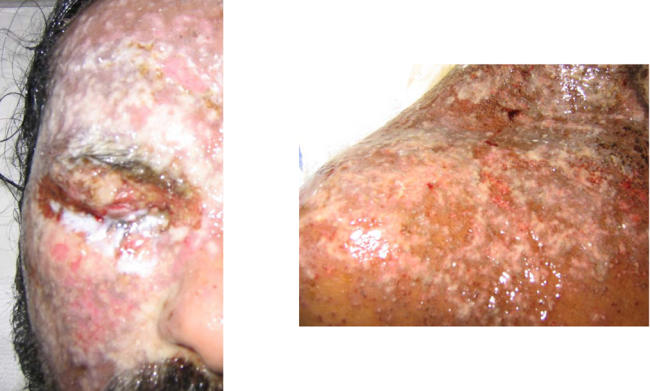
Appearance of Part of the Face and Shoulder at Presentation to Hospital There are multiple shallow ulcers with copious creamy exudates and some crusting. A few intact vesicles are also visible. The right eye was closed by exudates.

Because of a history of penicillin allergy, the patient was started on erythromycin. This treatment had no effect, and the rash extended. On day 4, he presented to the hospital accident and emergency department. He was systemically unwell, complaining of drenching sweats and rigors. On examination he was alert, he was pyrexial at 39.2 °C, his pulse was 100 bpm, and his blood pressure was 128/70 mm Hg. There was a widespread erythematous rash covering his face, chest, and arms, which was described as wet with a yellowish exudate. The rash extended around both eyes, which could not be opened (Figure 1).

The patient had a history of atopic eczema (since childhood), rhinitis, and asthma. He was taking inhaled corticosteroids for asthma and had last received a course of systemic steroids one year previously. There were no risk factors for HIV infection.

### Which Investigations Were Now Indicated?

In many patients with a rash, the morphology of the rash will lead to a provisional clinical diagnosis, and it may be sufficiently characteristic for no confirmatory tests to be needed. An example is the typical rash of shingles. There are two broad reasons for doing investigations in a patient presenting with fever and a rash. Firstly, specific tests—such as blood tests, cultures, or a skin biopsy—may be required to help establish the diagnosis. In this 33-y-old patient, the rash was thought likely to be a skin infection, and so the specific tests that were indicated included viral and bacterial cultures. A skin biopsy would have been considered if the diagnosis remained unclear and the patient was not responding to empirical antimicrobial therapy. Secondly, investigations are used to evaluate how ill the patient is and whether there is any organ involvement beyond the skin. In this case, we were most concerned about severe sepsis with a risk of organ failure.

Investigations showed the following: haemoglobin, 128 g/L (normal range, 135–170 g/L); white blood cell count, 9.9 × 10^9^/l (normal range, 3.5–12.5 × 10^9^/l); and differential white blood cell count, 90% neutrophils with a left shift. The patient was hyponatraemic (his sodium was 126 mmol/l [normal range, 135–145]), but renal and liver function tests were normal. C-reactive protein was 140 mg/l (normal level, <10 mg/l). Chest X ray was normal. Blood cultures and skin swabs were taken along with samples for viral culture and polymerase chain reaction (PCR).

### What Was the Provisional Diagnosis?

A provisional diagnosis of bacterially infected eczema was made, and, in view of his history of penicillin allergy, the patient was started on intravenous vancomycin. He was admitted and referred for dermatology, infectious diseases, and ophthalmology reviews.

On closer inspection of the rash, it was apparent that there were multiple small vesicles 1–2 mm in diameter and shallow ulcers over the face and periorbital region (Figure 1). There was extensive exudate on the skin of the eyelids of both eyes. It was not possible to open the right eye, but in the left eye, the cornea was clear and there was no evidence of disease.

### How Did This Information Affect the Likely Diagnosis?

In view of the characteristic picture and underlying eczema, the diagnosis was revised to one of severe eczema herpeticum. The patient denied any previous history of oral cold sores or genital ulceration. However, the severe degree of skin inflammation, high fever, raised C-reactive protein, and left-shifted neutrophils suggested co-existent bacterial sepsis.

### What Was the Management Approach?

High-dose intravenous aciclovir (10 mg/kg every 8 h) was started. Aciclovir inhibits thymidine kinase, which is an essential enzyme for several herpes viruses. Aciclovir has excellent activity against herpes simplex virus (HSV), and, although less potent, it is also effective against varicella zoster virus [1]. It has only weak activity against cytomegalovirus and Epstein-Barr virus [1].

This man had severe skin involvement with a high likelihood of complicating bacterial sepsis, which is most commonly due to Staphylococcus aureus [2]. Erythromycin had originally been prescribed, but increasing erythromycin resistance makes this a poor choice in many parts of the world. In view of his penicillin allergy, vancomycin was continued and ciprofloxacin added to broaden the antibacterial spectrum. Vancomycin disrupts the peptidoglycan bonding of the gram-positive bacterial cell wall and has no activity against gram-negative bacteria. Ciprofloxacin is a DNA gyrase inhibitor with excellent activity against many gram-negative bacteria, but it is less effective for gram-positive organisms. Aciclovir and chlorampenicol were administered topically to both eyes, and this treatment was successful in preventing corneal involvement.

Two separate sets of blood cultures subsequently grew S. aureus, which was resistant to penicillin, erythromycin, and clindamycin but sensitive to all other agents. Skin and eye swabs also grew S. aureus. HSV DNA was identified by PCR on fluid taken from the face. Viral cultures were positive for HSV-1. Serology taken on admission was positive for HSV-1 IgG.

### Progress

**Figure 2:**
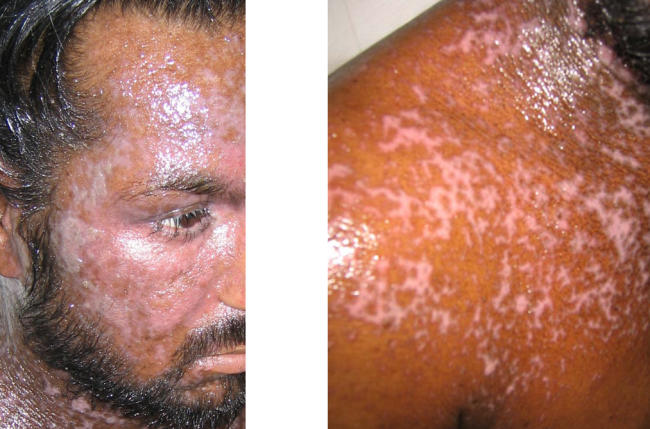
Appearance on Day 5 Most of the exudate has cleared, and all vesicles have desquamated. The eyes are now open, and the skin is beginning to heal.

**Figure 3:**
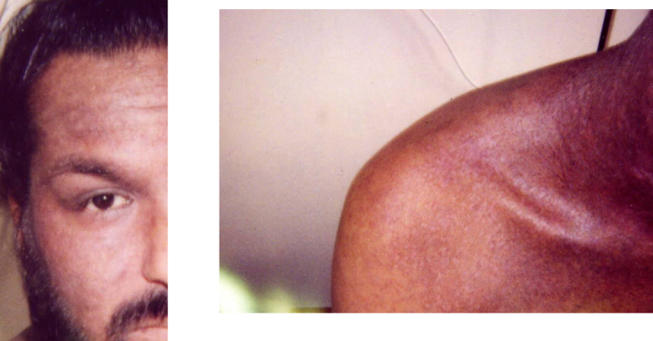
Appearance on Day 28 All ulcers have resolved, and healing is almost complete.

The patient rapidly defervesced, and the vesicles desquamated on day 6 (Figure 2). He was discharged after a total hospital stay of 16 d. He returned to the clinic 2 wk later, at which point the skin was healing well (Figure 3).

### What Complications Should Be Looked Out for?

In the acute stage of his presentation, our two main concerns for this patient were the extent of involvement of the eyes beneath the eyelids and the positive blood cultures for S. aureus. As mentioned above, the eyes were proactively managed to avoid corneal involvement. The S. aureus bacteraemia could have progressed to severe sepsis or led to metastatic spread of infection. Echocardiogram was normal with no evidence of endocarditis, and intravenous vancomycin was continued for a period of 14 d to minimize the risk of metastatic spread. Close attention to infection control procedures is essential to prevent hospital-acquired infection of the damaged skin or intravenous line sites.

## DISCUSSION

### Eczema Herpeticum

Kaposi's varicelliform eruption describes the widespread dermal infection caused predominantly by HSV-1 in patients with a variety of skin conditions [3]. It is most commonly seen in patients with pre-existing atopic eczema, in which case it is known as eczema herpeticum. Disease severity varies from mild, transient disease to a fulminating fatal disorder involving the visceral organs. Death is very rare in immunocompetent adults; fulminant disease is more likely in severely immunocompromised individuals. The incidence of eczema herpeticum is increasing, especially in the context of sexually transmitted disease [4]. Most cases are caused by reactivation of HSV rather than primary infection [3]. Our patient had no clinical history of infection with herpes, but HSV-1 was confirmed by both PCR and culture. The positive HSV-1 IgG titre suggests that this was reactivation of latent infection.

Prompt use of antiviral therapy is highly effective in controlling HSV replication. Aciclovir is the antiviral agent of choice and is usually given for 7 d. Patients with moderate to severe disease require admission for intravenous aciclovir. Oral aciclovir is effective for mild cases, which can be managed on an outpatient basis [5]. Bacterial superinfection is common in severe eczema herpeticum [2], and antibiotic therapy is required in severe cases. Valaciclovir (a pro-drug of aciclovir) and famciclovir are available in oral preparations and can be considered as alternative antivirals where oral therapy is an option [1]. In addition to the treatment of systemic infection, skin and eye lesions require very careful management, and specialised dermatology [6] and ophthalmology input are essential [7].

A proportion of patients may experience recurrent attacks of eczema herpeticum [4]. Recurrences are generally diagnosed and treated rapidly, as both the patients and their physicians have become familiar with the disease. With frequent recurrences, prophylactic aciclovir may be considered, although there are insufficient data to recommend its use routinely. Good control of eczema is also important to reduce the risk of recurrence [3].

### Which Patients with Atopic Eczema Are at Risk of Eczema Herpeticum?

A recent retrospective analysis of 100 cases of eczema herpeticum in patients with atopic ezcema highlighted some predisposing factors. The analysis found that patients with eczema herpeticum had developed atopic eczema earlier and had higher total serum IgE levels than control patients with atopic eczema alone [3]. In addition, 75% of the patients with eczema herpeticum had not received corticosteroid treatment in the 4 wk preceding onset of eczema herpeticum. The authors of the analysis concluded that most cases of eczema herpeticum occurred in patients with untreated atopic eczema. Thus, effective control of eczema is important to prevent eczema herpeticum. Such control is particularly important in our patient to prevent further attacks of eczema herpeticum.

### Making the Correct Diagnosis

The rapidly progressive, widespread, crusted papules, vesicles, and erosions in a patient with atopic eczema are characteristic, and an experienced physician who has seen eczema herpeticum before will make a rapid clinical diagnosis. However, some patients present with atypical or purely crusted lesions and no obvious vesicles, making a clinical diagnosis more difficult. Thus, a high index of clinical suspicion is required, and both viral and bacterial cultures are essential when a patient with atopic eczema presents with a spreading vesicular or crusting rash [4]. Rapid confirmation of cutaneous HSV can be achieved with immunofluorescent techniques or PCR, but these are not yet available in many centres [8].

### Smallpox Vaccination

A similar and potentially fatal disease may follow smallpox vaccination [9,10]. Until recently, this was only of academic interest, given the global eradication of smallpox. Recent events have raised the spectre of smallpox returning, however, and some countries have begun stockpiling vaccine and inoculating key health-care workers. Smallpox vaccination involves the intradermal inoculation of live attenuated variola virus by scarification, and the site may shed live virus for 2–3 wk until the lesion heals. In a person with eczema, the variola virus can cause widespread eczema vaccinatum. As the variola virus is shed into the environment from the vaccine site, it can also spread to close contacts with eczema, which can lead to secondary cases of eczema vaccinatum. This risk is greatly diminished with the use of an appropriate occlusive dressing to the vaccination site. It is reassuring that, so far, no cases of eczema vaccinatum have been reported in the United States following the recent smallpox vaccination programme, but this disease may well return if there is need for more extensive smallpox vaccination.[Boxed-text pmed-0010017-box1]

**Key Learning Points**

- Eczema herpeticum is an uncommon but potentially fatal disorder that can present to primary care practitioners or to the emergency department.
- Diagnostic delay is common and may increase morbidity.
- First-line therapy is with acyclovir; intravenous high-dose therapy is required in severe cases.
- Patients should be referred to a dermatologist for specialist care and follow-up to optimise eczema management, so that recurrence is less likely.
- Intensive nursing care is indicated to minimise scarring and promote rapid healing.
- If the periorbital region is involved, refer urgently to an ophthalmologist.

## Abbreviations

HSV: herpes simplex virus
PCR: polymerase chain reaction
